# Use of Tobacco and Cannabis Following State-Level Cannabis Legalization

**DOI:** 10.1001/jamanetworkopen.2025.20093

**Published:** 2025-07-11

**Authors:** Andrew S. Hyatt, Lindsay Overhage, Benjamin Lê Cook

**Affiliations:** 1 Health Equity Research Lab, Cambridge Health Alliance, Cambridge, Massachusetts; 2 Department of Psychiatry, Harvard Medical School, Boston, Massachusetts

## Abstract

**Importance:**

Cannabis use has been shown to modestly increase shortly after legalization of recreational use, but little is known about longer-term changes in cannabis and tobacco use.

**Objective:**

To assess the association of cannabis legalization and commercialization with the use of cannabis, cigarettes, and electronic nicotine delivery systems (ENDS) in the 5 years after legalization in the US.

**Design, Setting, and Participants:**

The Population Assessment of Tobacco and Health (PATH) study, a nationally representative longitudinal cohort study of adults 18 years or older in the US, was used for a retrospective, difference-in-differences analysis of panel data from January 2013 to December 2022. Data were analyzed from May 8, 2024, to April 20, 2025.

**Exposures:**

State-level recreational cannabis legalization (RCL) and opening of retail outlets with 5 years of follow-up.

**Main Outcomes and Measures:**

Percentage point changes in 30-day use of cannabis, cigarettes, and ENDS in states with legalization compared with control states, aggregated over 5 years and then broken down into the periods before and after retail outlets opened.

**Results:**

Among the 171 257 observations from 55 406 individuals included in the analysis (50.9% female; mean [SD] age, 37.97 [17.69] years), RCL was associated with an increase of cannabis use by 3.28 (95% CI, 2.29-4.27) percentage points and ENDS use by 1.39 (95% CI, 0.44-2.35) percentage points compared with control states. There was no differential change in cigarette use (−0.99 [95% CI, −2.25 to 0.27] percentage points). For cannabis, this change was greater after retail outlets opened (3.74 [95% CI, 2.65-4.82] percentage points) compared with before (1.17 [95% CI, 0.13-2.20] percentage points). There were no large changes in ENDS or cigarette use associated with the opening of retail outlets.

**Conclusions and Relevance:**

In this longitudinal cohort study, RCL was associated with more cannabis and ENDS use after 5 years and no significant change in cigarette use. Furthermore, cannabis use increased over time, underscoring the importance of studying commercialization policy going forward. Based on the present results, it is unlikely RCL has been associated with a large increase in cigarette use, but increases in use of cannabis and vaped nicotine bear close monitoring as retail cannabis rapidly expands.

## Key Points

**Question:**

Is legalizing cannabis associated with use of cannabis, cigarettes, and electronic nicotine delivery systems (ENDS)?

**Findings:**

In this cohort study using difference-in-differences methods to analyze 171 257 observations from 55 406 participants, legalization was associated with increased cannabis use and ENDS use. There was no association with cigarette use.

**Meaning:**

Cannabis and ENDS use increased after cannabis legalization, but there was no evidence of a halt or reverse in the decline in cigarette use.

## Teaser

This cohort study assesses whether there were changes in the use of cannabis, cigarettes, and electronic nicotine delivery systems among US adults during 5 years following cannabis legalization.

## Introduction

Cannabis is the most widely used illicit substance worldwide,^1^ and while it has fewer harms than alcohol and tobacco,^2,3^ it has been associated with many negative sequelae, including motor vehicle accidents,^4,5^ cannabis use disorder,^6^ and mental health problems.^7,8,9^ Recreational cannabis legalization (RCL) has spread rapidly throughout the US, with 24 states having legalized retail cannabis as of December 2024.^10^ Early research has documented modest increases in adult cannabis use after legalization over 2 to 3 years^11,12,13,14,15^ but has not assessed longer follow-up and whether these effects change as the different phases of RCL roll out. Legalization of possession is associated with decreased risk perceptions and increased use,^16^ and the opening of commercial outlets and subsequent increases in advertising and availability may be associated with further increases in use.^17,18^ It is thus crucial to evaluate the effects of legalization over time and to account for commercialization effects in evaluating policy.

Tobacco is the leading cause of preventable mortality in the US,^19^ and there is little research on how RCL affects tobacco use. Cannabis and tobacco are frequently used together,^20,21^ and use of cannabis is associated with higher rates of initiation of tobacco use and lower quit rates, suggesting possible complementarity.^22,23^ On the other hand, medical cannabis legalization may have led to small decreases in tobacco use,^24^ suggesting that RCL could also lead to decreased tobacco use. Additionally, while smoking rates have fallen, use of electronic nicotine delivery systems (ENDS) has dramatically increased over the past 15 years.^25,26^ Vape products are popular for the delivery of both nicotine and cannabis, and there is ongoing debate about the relative health merits of ENDS use as a harm reduction tool for tobacco users^27^ vs serving as a pathway for the initiation of combustible tobacco use.^28,29^

The few studies examining the association of RCL with tobacco use^30,31,32^ have been mixed. One study found no association up to 2016,^30^ a national survey found RCL was associated with decreased cigarette only use but with increased co-use of cannabis and combustible tobacco,,^31^ and a third study found that RCL was associated with decreased ENDS and combustible tobacco use after 3 years.^32^

We used difference-in-differences (DiD) methods^33^ applied to the Population Assessment of Tobacco and Health (PATH) study population to assess changes in the use of cannabis, cigarettes, and ENDS in the 5 years following cannabis legalization. We hypothesized that RCL would be associated with increased cannabis use, with higher estimates as policy implementation progressed, and that RCL would be associated with less ENDS and cigarette use.

## Methods

### Data

This longitudinal cohort study analyzed restricted use data of adults 18 years and older from the PATH study. PATH is an ongoing nationally representative US-based longitudinal cohort study conducted by the National Institute on Drug Abuse in partnership with the Center for Tobacco Products of the US Food and Drug Administration, and it collects individual-level data on the use of tobacco products, substance use, and demographics.^34^ Data collection occurred in 7 waves from January 2013 to December 2022, and occurred as planned with the exception of 2020, when no surveys were collected due to the COVID-19 pandemic. Each wave contained approximately 25 000 to 30 000 responses collected over 12 to 15 months. All participants provided written informed consent and received a participant incentive at the time of the survey. This secondary data analysis was deemed exempt from review by the Institutional Review Board of the Cambridge Health Alliance because the research was a secondary data analysis of in which the identity of original participants could not be readily ascertained. This study followed the Strengthening the Reporting of Observational Studies in Epidemiology (STROBE) reporting guideline.

### Exposures

The primary exposure of interest was state-level RCL, defined as the effective date of a law to legalize adult possession of nonmedical cannabis. The secondary exposure was the opening of commercial retail cannabis outlets, operationalized as the date of first legal sale in a state. Both dates were extracted from the National Institute on Alcohol Abuse and Alcoholism Alcohol Policy Information System^35^; in case of ambiguity, supplemental information was obtained from the Marijuana Policy Project.^10^ Dates of legalization and commercialization as well as which states were in the treatment, control, and excluded groups can be found in Table 1.

**Table 1.** Dates of Cannabis Legalization and First Retail Sales by State

State	Cannabis legal date	Cannabis retail date	Inclusion
Alabama	NA	NA	Control
Alaska	2/24/2015	10/29/2016	Excluded^a^
Arizona	11/1/2020	01/22/2021	Treatment
Arkansas	NA	NA	Control
California	11/9/2016	01/01/2018	Treatment
Colorado	12/10/2012	01/01/2014	Excluded^b^
Connecticut	7/1/2021	NA	Treatment
Delaware	NA	NA	Excluded^a^
Florida	NA	NA	Control
Georgia	NA	NA	Control
Hawaii	NA	NA	Control
Idaho	NA	NA	Excluded^a^
Illinois	01/01/2020	01/01/2020	Treatment
Indiana	NA	NA	Control
Iowa	NA	NA	Control
Kansas	NA	NA	Excluded^a^
Kentucky	NA	NA	Control
Louisiana	NA	NA	Control
Maine	1/30/2017	10/09/2020	Treatment
Maryland	NA	NA	Control
Massachusetts	12/15/2016	11/20/2018	Treatment
Michigan	11/6/2018	12/01/2019	Treatment
Minnesota	NA	NA	Control
Mississippi	NA	NA	Control
Missouri	12/1/2022	NA	Excluded^c^
Montana	1/1/2021	01/01/2022	Treatment
Nebraska	NA	NA	Control
Nevada	1/1/2017	07/01/2017	Treatment
New Hampshire	NA	NA	Control
New Jersey	2/1/2021	04/21/2022	Treatment
New Mexico	6/1/2021	04/01/2022	Excluded^a^
New York	3/1/2021	12/29/2022	Treatment
North Carolina	NA	NA	Control
North Dakota	NA	NA	Excluded^a^
Ohio	NA	NA	Control
Oklahoma	NA	NA	Control
Oregon	6/30/2015	10/01/2015	Treatment
Pennsylvania	NA	NA	Control
Rhode Island	5/1/2022	12/01/2022	Excluded^a^^,^^c^
South Carolina	NA	NA	Control
South Dakota	NA	NA	Excluded^a^
Tennessee	NA	NA	Control
Texas	NA	NA	Control
Utah	NA	NA	Control
Vermont	7/1/2018	10/01/2022	Excluded^a^
Virginia	7/1/2021	NA	Treatment
Washington	12/6/2012	07/08/2014	Excluded^b^
Washington, DC	2/26/2015	NA	Excluded^a^
West Virginia	NA	NA	Control
Wisconsin	NA	NA	Control
Wyoming	NA	NA	Excluded^a^

Abbreviation: NA, not applicable.

^a^ State was not included in primary Population Assessment of Tobacco and Health sample.

^b^ State legalized cannabis use before the study period.

^c^ State legalized cannabis use at the end of the study period.

PATH restricted use files contain the month and year of interview as well as current state of residence, allowing the linkage of individual responses to state cannabis policy at the time of the interview. Individuals were coded as exposed to the policy if their response was in the year of the policy change or any year afterward. Responses during the year of policy change (year 0) were excluded from aggregate analyses as policy changes occurred throughout the calendar year, and any individual response may have occurred before or after the policy change. Follow-up continued to 5 years after legalization.

### Outcomes

The 3 outcomes of interest were past 30-day use of cannabis, cigarettes, and ENDS. Use was assessed with individual questions asking, “In the past 30 days have you used [substance], even once or twice?” A response of yes to this question was coded as positive in a binary indicator variable.

### Covariates

Time-varying covariates across time and state that could confound DiD models were indoor workplace smoking bans (tobacco and ENDS), inclusion of electronic nicotine products in indoor smoking bans (ENDS), and taxes on cigarettes and ENDS products. Data on indoor smoking bans were taken from the American Nonsmokers’ Rights Foundation,^36^ state-level cigarette taxes from the State Tobacco Activities Tracking and Evaluation system of the Centers for Disease Control and Prevention,^37^ and ENDS taxes taken from published standardized rates across the 50 states.^38^ Individual covariates included race and ethnicity, receipt of state or federal antipoverty assistance, gender, and age as self-reported by participants. Race and ethnicity were characterized as Hispanic, non-Hispanic Asian, non-Hispanic Black, non-Hispanic White, and other (including American Indian or Alaska Native, Native Hawaiian or Other Pacific Islander, or multiracial) and were included because of the disparate impact of tobacco and drug control policies on minoritized populations.

### Statistical Analysis

First, we characterized the sample overall by individual covariates and overall rates of our 3 outcomes. Next, rates of cannabis, cigarette, and ENDS use were graphed during the study period, with states that legalized cannabis in a given year grouped together and compared with states that never legalized cannabis.

In accordance with DiD best practices for evaluating time-varying interventions,^39,40,41^ we used Callaway-Sant’Anna DiD models to assess RCL during a 5-year period.^42^ DiD can produce valid estimates under 2 primary assumptions.^43^ First, in the parallel trends assumption that, in the present study, states that do and do not enact cannabis policy changes (treatment) would have similar trends in cannabis use unless a policy change was enacted. Second, the no anticipation assumption holds that there is no change in states that have legalized cannabis ahead of the policy change. To assess these assumptions, we first tested for parallel trends with Wald tests in all periods before treatment, then graphed event study plots for each outcome with no significant prelegalization policy treatment estimates being consistent with parallel trends. If diversions were found, event study plots were examined by cohort to identify which legalization cohorts were contributing to nonparallel prelegalization trends. If nonparallel trends could not be established by conditioning on covariates, this cohort was removed from the analysis.

After testing the validity of the aforementioned assumptions, we then reported DiD results conditioned on covariates. States that never legalized cannabis during the study period served as controls. Results were reported as percentage point changes. Robust SEs were clustered within each individual to account for autocorrelation of each individual’s responses. We then reported results from event-study plots to assess changing policy associations over time.^44^

To assess changes following legalization of possession compared with the opening and spread of retail outlets, a 2-stage DiD model was constructed. First, analyses were rerun including only the years of legalization before retail outlets opened, then run a second time including only the years after retail outlets opened to assess changes associated with wider spread of commercialization. All data analyses were undertaken between May 8, 2024, and April 20, 2025, in Stata, version 18 (StataCorp LLC).

We implemented several checks to probe the robustness of our findings. First, to ensure results were robust to differing years of follow-up available depending on the year of legalization, we conducted an analysis restricting DiD estimates to states legalizing cannabis in 2017 or earlier to ensure 5 years of follow-up. Second, we restricted analyses to individuals 21 years or older, the age at which possession and purchase of cannabis is legalized in all states. Third, we implemented leave-one-out robustness checks by dropping each state with legalization in turn and rerunning analyses to ascertain whether any particular states were disproportionately influencing results. Fourth, to reduce the risk of poorly matched controls, we removed any control state that decriminalized cannabis from the analysis. Fifth, to assess changes in more frequent cannabis use, we reran the analysis using weekly or greater cannabis use as the outcome. Sixth, we reran models adding American Community Survey^45^ state-level estimates of median age and percentage of the population with a college degree as covariates given confounding effects in prior studies.^30^ Last, we ran a standard 2-way fixed-effects DiD model to enable comparisons with previous work using older DiD models.

## Results

### Sample

A total of 56 090 unique individuals participated in at least 1 wave of the PATH study, and there were 218 262 unique responses of adults 18 years or older. Responses missing individual covariates, obtained after an individual moved, from states with no prelegalization or postlegalization data, and from more than 5 years after RCL were excluded, leaving a final analytic sample of 171 257 observations from 55 406 individuals. eFigure 1 in Supplement 1 provides a flow diagram of the sample.

### Descriptive Results and Investigation of Assumptions

A total of 171 257 observations from 55 406 individuals were included in the analysis, including 50.9% female and 49.1% male observations (mean [SD] age, 37.97 [17.69] years). In terms of race and ethnicity, 5.2% of observations were from Hispanic participants; 2.6%, non-Hispanic Asian; 15.4%, non-Hispanic Black; 56.4%, non-Hispanic White; and 20.4%, other. The sample is described by demographic characteristics and outcomes in Table 2. Descriptive trends in cannabis, tobacco, and ENDS use by year and year of legalization were graphed (eFigures 2-4 in Supplement 1). Wald tests found no evidence of diverging prelegalization trends in cannabis (χ^2^ = 9.38; *P* = .86) and ENDS (χ^2^ = 20.29; *P* = .16) use, but did for cigarette use (χ^2^ = 28.42; *P* = .02). Next, event study plots were graphed, supporting parallel trends for cannabis and ENDS use. We identified a deviation from parallel trends in the 2016 cohort for cigarettes only (eFigure 5 in Supplement 1). This cohort was removed from the analysis. When adjusted models were rerun, there was no evidence of divergent parallel trends (χ^2^ = 15.87; *P* = .25), and the event study plot was consistent with this finding.

**Table 2.** Description of Analytic Sample^a^

Demographic characteristic	Responses, No. (%)	30-d Use, %		
		Cannabis	Cigarette	ENDS
Overall rates	NA	19.1	33.7	14.0
Race and ethnicity				
Hispanic	35 159 (20.5)	18.1	26.8	13.2
Non-Hispanic Asian	4504 (2.6)	10.5	16.9	9.4
Non-Hispanic-Black	28 929 (16.5)	21.7	35.6	9.9
Non-Hispanic White	94 756 (55.3)	18.4	35.9	15.1
Other^b^	10 262 (5.0)	27.1	38.6	13.2
Gender reported				
Female	87 204 (50.9)	17.2	32.5	13.1
Male	84 053 (49.1)	21.1	34.9	13.2
Age, y				
Mean (SD)	37.97 (17.69)	NA	NA	NA
Range	18-90	NA	NA	NA
Age range, y				
18-24	56 383 (32.9)	24.5	22.5	19.4
25-34	34 174 (20.0)	24.1	38.9	16.9
35-44	22 742 (13.3)	18.5	42.3	13.0
45-54	21 095 (12.3)	14.5	43.9	10.3
55-64	19 840 (11.6)	12.7	42.7	7.7
≥65	17 024 (9.9)	5.4	25.1	3.7
Received state or federal financial assistance in past 12 mo				
No	135 516 (79.1)	17.8	29.5	13.6
Yes	35 741 (20.9)	25.2	47.8	15.6

Abbreviations: ENDS, electronic nicotine delivery systems; NA, not applicable.

^a^ Includes 171 257 observations from 55 406 individuals.

^b^ Includes American Indian or Alaskan Native, Native Hawaiian or Other Pacific Islander, or Multiracial.

### Overall DiD Estimates

**Figure 1.**
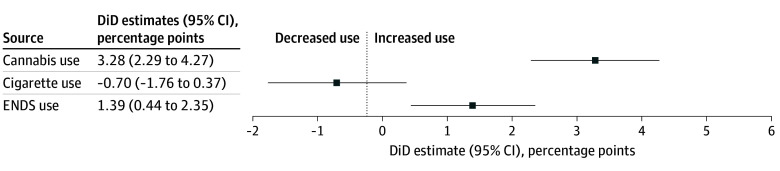
Difference-in-Differences (DiD) Estimates of the Association of Recreational Cannabis Legalization (RCL) With 30-Day Substance Use All DiD estimates adjusted for race and ethnicity, gender, age, and receipt of past-year state or federal financial assistance. Cigarette models adjusted for cigarette taxes and exposure to indoor clean air laws by state and year. Electronic nicotine delivery systems (ENDS) models adjusted for ENDS taxes and inclusion of ENDS in indoor clean air laws.

**Figure 2.**
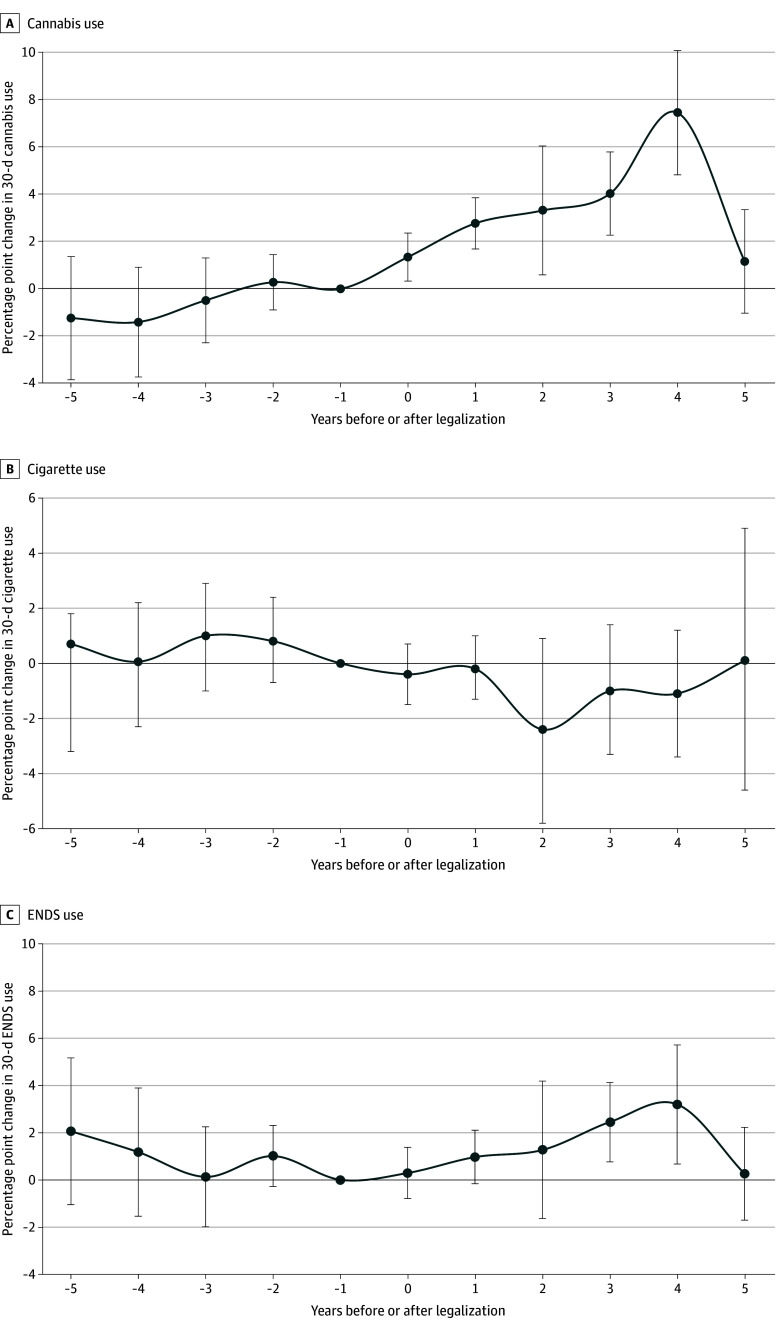
Event-Study Plots of the Association of Recreational Cannabis Legalization With 30-Day Substance Use All event-study plots display estimates in a given year normalized to the year before policy implementation (year −1) with 95% CIs (error bars). Year 0 is the year cannabis legalization went into effect in a given state. All models were conditioned on the same covariates as noted in Figure 1.

All DiD estimates are displayed in Figure 1. The aggregate 5-year DiD estimate of the association of RCL with 30-day cannabis use was positive (3.28 [95% CI, 2.29-4.27] percentage points; *P* < .001). Event-study estimates showed increasing use over time through year 4 of the study period before dropping to a positive but nonsignificant rate of 1.16 (95% CI, −1.03-3.35) percentage points in year 5 (Figure 2A).

The aggregate 5-year DiD estimate showed no association of RCL with 30-day cigarette use (−0.99 [95% CI, −2.25 to 0.27] percentage points; *P* = .12) and in all years post treatment (Figure 2B). The aggregate 5-year DiD estimate of RCL associated with 30-day ENDS use was small but positive (1.39 [95% CI, 0.44-2.35] percentage points; *P* = .004). Event-study estimates showed associations in only years 3 and 4 (Figure 2C).

### DiD Estimates of Legalization vs Commercialization

Legalization was associated with a small increase in cannabis use (1.17 [95% CI, 0.13-2.20] percentage points; *P* = .03), but commercialization was associated with a larger increase in cannabis use (3.74 [95% CI, 2.65-4.82] percentage points; *P* < .001). For ENDS, estimates were similar before (1.41 [95% CI, 0.26-2.56] percentage points; *P* = .02) and after commercialization (1.21 [95% CI, 0.16-2.27] percentage points; *P* = .02). For cigarettes, estimates were also similar before (−0.25 [95% CI, −1.35 to 0.85] percentage points; *P* = .66) and after (−0.64 [95% CI, −1.73 to 0.45] percentage points; *P* = .25) commercialization.

### Sensitivity Analyses and Robustness Checks

First, restricting the analysis to only states with at least 5 years of follow-up produced similar results, with a smaller point estimate for cannabis. Second, restricting analyses to individuals 21 years or older produced similar results to the main analyses. Third, leave-one-out robustness checks revealed that for cannabis, dropping California raised the DiD estimate and normalized the event study in year 5, while dropping Michigan lowered the DiD estimate. For ENDS, dropping Michigan or California reduced the significance of the ENDS estimate. Fourth, removing control states that decriminalized cannabis did not change results. Fifth, 2-way fixed-effects estimates were similar for cannabis and tobacco, and negative but not significant for ENDS. Sixth, RCL did not have an association with weekly or more frequent cannabis use. Seventh, adding controls for state-level age distribution and educational attainment did not significantly alter results. Full results are available in eTables 1 and 2 in Supplement 1.

## Discussion

At 5 years, this retrospective longitudinal cohort study found that RCL was associated with an increase of 3.28 percentage points in cannabis use, 1.39 percentage points in ENDS use, and no significant changes in cigarette use. To our knowledge, this is the first study to evaluate RCL to 5 years and one of the first to investigate the associations of RCL with tobacco and ENDS use.

Consistent with prior literature,^11,12,13,14,15,32^ cannabis use rose after RCL. Notably, this result was larger than that seen in these studies, suggesting that longer follow-up allowed us to capture more complete policy effects encompassing spread of retail outlets, market saturation, and changes in social norms. Consistent with this, event-study estimates showed positive and increasing changes in years 1 to 4, and RCL was associated with greater changes in use after dispensaries were open than before. Prior literature has often not taken into account the lagged effects of commercialization,^46,47^ which our longer follow-up and specific analyses allowed us to investigate. Additionally, the use of longitudinal data allowed us to exclude population movement and demographic shifts between states as a source of change.^48^ The drop in year 5 was unexpected and could be partially an artifact of the COVID-19 pandemic, as no surveys were collected in 2020, meaning not all cohorts had follow-up in year 5. Additionally, it is possible that several years after legalization, a ceiling effect was reached, whereas lower baseline rates in states without legalization had further room to continue increasing. Sensitivity analyses also suggested this result was sensitive to the inclusion of large states with long follow-up. Further research should be able to clarify changes at longer time horizons. Finally, sensitivity analyses did not find a significant change in weekly cannabis use, and future research can investigate whether increases in past-month use translate to lagged increases in heavier use.

There was no evidence that RCL was associated with cigarette use, contrasting with prior results showing a drop in cigarette use after legalization.^31^ A more recent analysis found evidence of a lagged decrease in cigarette use in the National Survey on Drug Use and Health but not in PATH,^32^ suggesting some differences in longitudinal vs cross-sectional policy evaluations. The present study provides evidence against the hypothesis that cannabis legalization leads to an increase in cigarette use, possibly due to the rise of noncombustible cannabis use (eg, edibles, vapes), as these means of consumption have been associated with less tobacco co-use.^49^ Finally, the unadjusted trends in cigarette use during the study period showed sharp declines across all states, which could overwhelm small changes due to a policy.

Our study found RCL was associated with a modest but significant increase in ENDS use, the opposite of what was found in another recent study showing less ENDS use after RCL.^32^ A sensitivity analysis using a similar but not identical 2-way fixed-effects model to the previous study revealed no significant decrease in ENDS use, suggesting that the choice of DiD estimator can alter the direction of results reported. ENDS use rising after RCL could be due to the rapid rise of vapes as a preferred delivery device for both nicotine and cannabis, especially among younger people,^50^ and adds to the evidence base suggesting ENDS and cannabis are complements instead of substitutes.^51^ The public health effects of this are unclear. On one hand, the co-use of nicotine and tetrahydrocannabinol in the same vape device was linked to an outbreak of e-cigarette, or vaping, product use–associated lung injury in 2019 to 2020 that was responsible for 68 deaths and almost 3000 hospitalizations.^52^ Additionally, there is some evidence that ENDS use in adolescence and young adulthood is associated with later initiation of combustible tobacco use.^53^ However, through 5 years of follow-up, there was no evidence of this, and despite the rapid rise in ENDS use among youths and young adults, there has not yet been any uptick in combustible tobacco use in this group.^54^

### Limitations

This study has limitation. DiD estimates for cannabis use were the most robust given the tight link between the policy and outcome and the robust support for the no anticipation and parallel trends assumptions. Support for these assumptions was more tenuous for cigarette use. While removing the 2016 cohort resulted in a nonsignificant prelegalization trend estimate, this could be due in part to the underpowered nature of such tests. ENDS had parallel prelegalization trends, but results were less robust when particular states were removed, suggesting significant policy heterogeneity and less uniform results. Next, in every state aside from Ohio, RCL was preceded by medical cannabis legalization (MCL), which could affect prelegalization trend estimates and make it harder to disentangle RCL from MCL using DiD models. However, past studies have found association with MCL laws,^55^ somewhat mitigating this concern. Additionally, although 5 years is more follow-up than in previously published studies, the lag between legalization and the first retail outlets opening and the often years-long lag between one outlet opening and wider spread mean that this duration of follow-up could be underestimating how widespread legal retail cannabis availability changes use. Finally, while the PATH study is nationally representative, it is not necessarily representative of each state, which could somewhat limit external validity.

## Conclusions

In this cohort study with 5 years of follow-up, RCL and subsequent commercialization had a positive association with cannabis use and ENDS use and no association with cigarette use. These results can inform the development of cannabis and tobacco control policies in states that have legalized or are considering legalizing cannabis and demonstrates the need for increased surveillance of use of electronic nicotine products and their effects, especially in vulnerable subpopulations such as individuals with or at risk for substance use and mental health problems.

## Notes

Supplement 1. **eFigure 1.** CONSORT Diagram on the Construction of the Analytic Sample **eFigure 2.** Descriptive Trends of Self-Reported Cannabis Use by Year in Legalization Cohort Compared With Controls **eFigure 3.** Descriptive Trends of Self-Reported Cigarette Use by Year in Legalization Cohort Compared With Controls **eFigure 4.** Descriptive Trends of Self-Reported ENDS Use by Year in Legalization Cohort Compared With Controls **eFigure 5.** RCL Association by Legalization Cohort With 30-day Cigarette Use **eTable 1.** Sensitivity Analyses **eTable 2.** Leave-One-Out Robustness Checks

Supplement 2.
Data Sharing Statement
