# Supplementary material for: Use of Tobacco and Cannabis Following State-Level Cannabis Legalization
Source: JAMA Netw Open. 2025 Jul 11;8(7):e2520093. doi: 10.1001/jamanetworkopen.2025.20093 (PMC12254894; doi:10.1001/jamanetworkopen.2025.20093)
Supplement: Supplement 1. — eFigure 1. CONSORT Diagram on the Construction of the Analytic Sample eFigure 2. Descriptive Trends of Self-Reported Cannabis Use by Year in Legalization Cohort Compared With Controls eFigure 3. Descriptive Trends of Self-Reported Cigarette Use by Year in Legalization Cohort Compared With Controls eFigure 4. Descriptive Trends of Self-Reported ENDS Use by Year in Legalization Cohort Compared With Controls eFigure 5. RCL Association by Legalization Cohort With 30-day Cigarette Use eTable 1. Sensitivity Analyses eTable 2. Leave-One-Out Robustness Checks [file jamanetwopen-e2520093-s001.pdf]

Supplementary Online Content

Hyatt AS, Overhage L, Cook BL. Use of tobacco and cannabis following state-level cannabis legalization. *JAMA Netw Open.* 2025;8(7):e2520093. doi:10.1001/jamanetworkopen.2025.20093

eFigure 1. CONSORT Diagram on the Construction of the Analytic Sample
eFigure 2. Descriptive Trends of Self-Reported Cannabis Use by Year in Legalization Cohort Compared With Controls
eFigure 3. Descriptive Trends of Self-Reported Cigarette Use by Year in Legalization Cohort Compared With Controls
eFigure 4. Descriptive Trends of Self-Reported ENDS Use by Year in Legalization Cohort Compared With Controls
eFigure 5. RCL Association by Legalization Cohort With 30-day Cigarette Use
eTable 1. Sensitivity Analyses
eTable 2. Leave-One-Out Robustness Checks

This supplementary material has been provided by the authors to give readers additional information about their work.

© 2025 Hyatt AS et al. *JAMA Network Open*.

Figures

eFigure 1. CONSORT Diagram on the Construction of the Analytic Sample

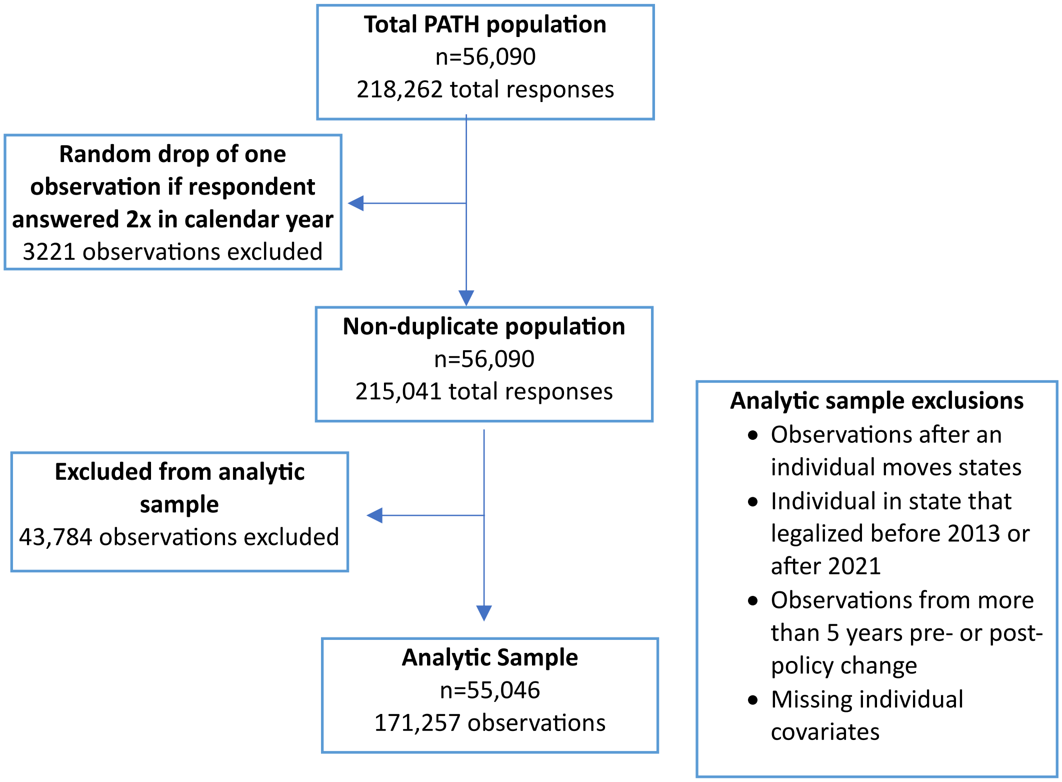

© 2025 Hyatt AS et al. *JAMA Network Open*.

eFigures 2. Descriptive Trends of Self-Reported Cannabis Use by Year in Legalization Cohort Compared With Controls

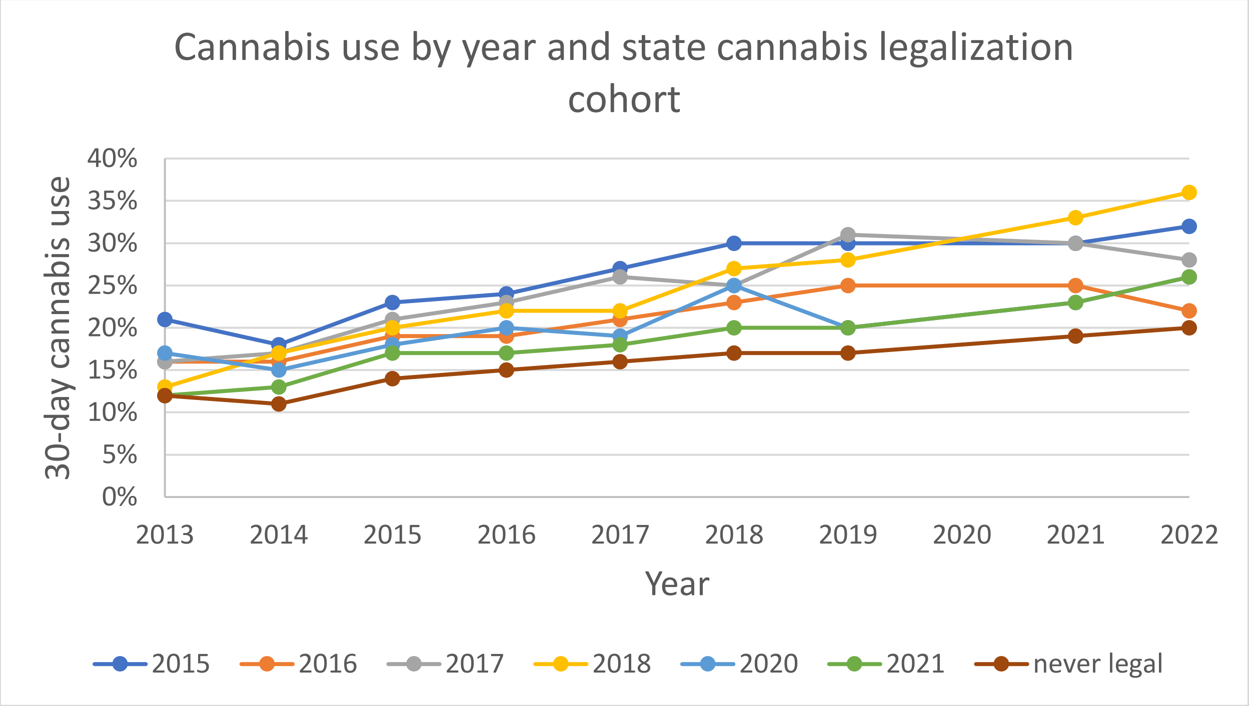

eFigure 3. Descriptive Trends of Self-Reported Cigarette Use by Year in Legalization Cohort Compared With Controls

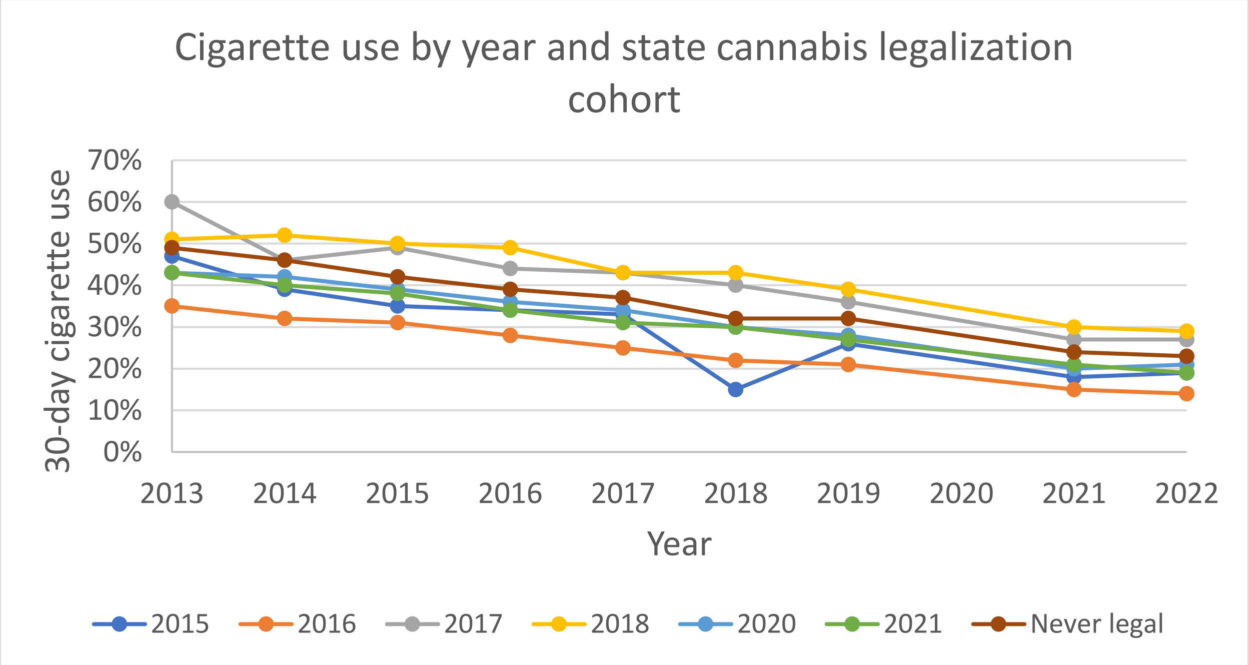

© 2025 Hyatt AS et al. *JAMA Network Open*.

eFigure 4. Descriptive Trends of Self-Reported ENDS Use by Year in Legalization Cohort Compared With Controls

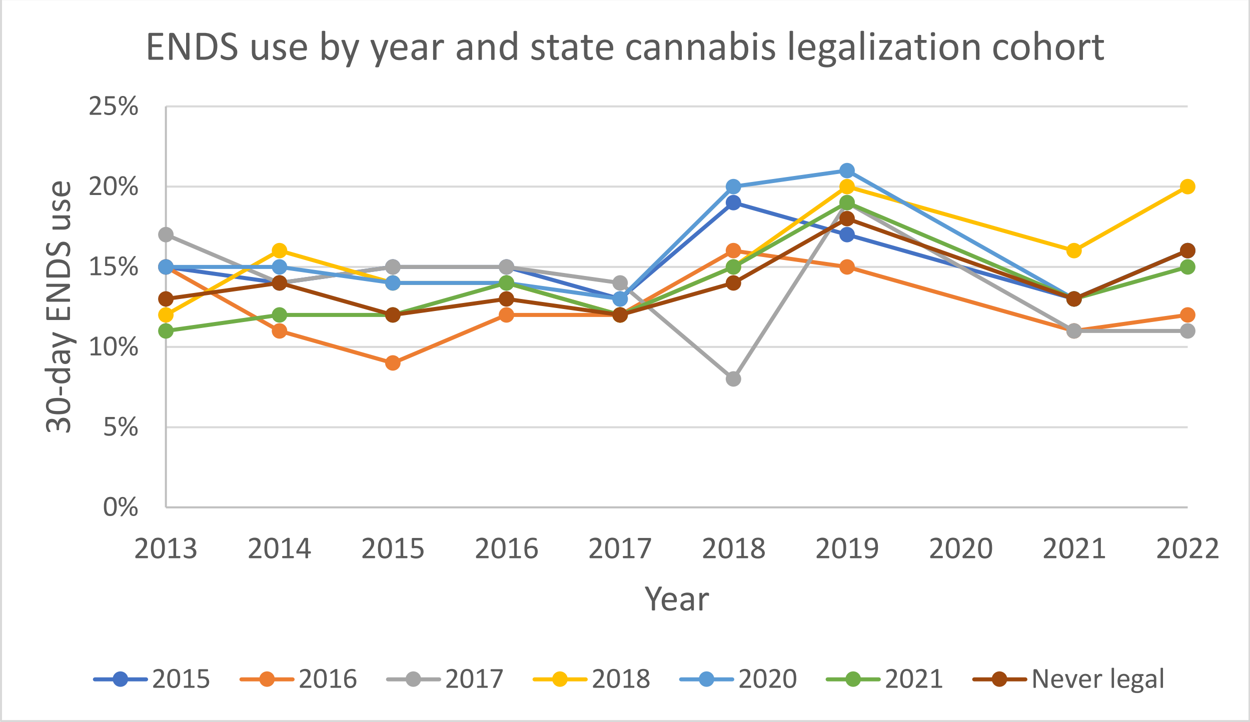

eFigure 5. RCL Association by Legalization Cohort With 30-day Cigarette Use

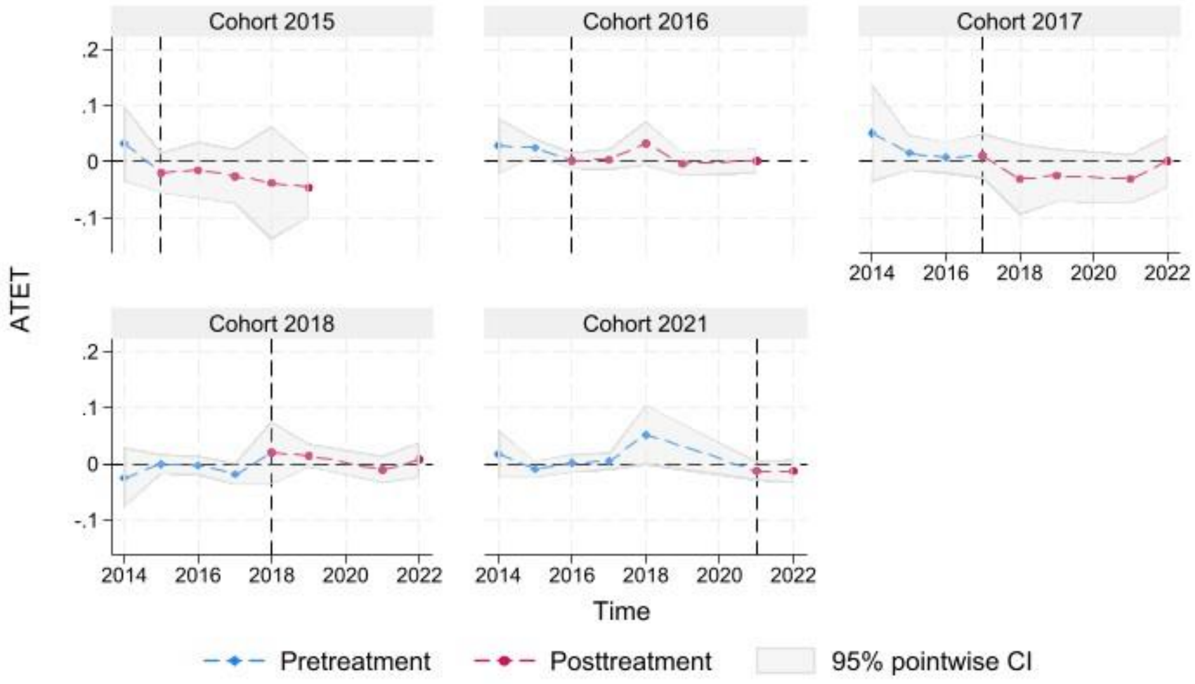

© 2025 Hyatt AS et al. *JAMA Network Open*.

eTable 1. Sensitivity Analyses

Sensitivity analyses and outcomes	DiD Estimates in percentage points (95% CI)	P value
**Restricting to states with 5 years of follow-up**		
Cannabis	2.05pp (0.63-3.47pp)	p=0.004
Cigarettes	-0.23pp (-1.57-1.27pp)	p=0.84
ENDS	1.50pp (0.16-2.84pp)	p=0.029
**Restricting to respondents aged 21+**		
Cannabis	3.40pp (2.36-4.45)	p<0.001
Cigarettes	-1.0pp (-2.36-0.35)	p=0.15
ENDS	1.21pp (0.20-2.21)	p=0.018
**Removing control states that decriminalized during study period**		
Cannabis	3.37pp (2.38-4.36pp)	p<0.001
Cigarettes	-0.38pp (-1.38-0.63pp)	p=0.46
ENDS	1.10pp (0.15-2.05pp)	p=0.023
**Changing outcome to weekly + cannabis use**		
Cannabis	0.47pp (-0.33-1.28pp)	p=0.25
**Adding ACS state-level education and median age controls**		
Cannabis	3.27pp (2.21-4.32pp)	p<0.001
Cigarettes	-0.60pp (-1.67-0.47pp)	p=0.27
ENDS	1.5pp (0.46-2.57pp)	p=0.005
**Alternate DiD model: standard two way fixed effects (TWFE)**		
Cannabis	3.41pp (2.63-4.20pp)	p<0.001
Cigarettes	-0.7pp (-1.58-0.12pp)	p=0.092
ENDS	-0.50pp (-1.39-0.40pp)	p=0.28

© 2025 Hyatt AS et al. *JAMA Network Open*.

eTable 2. Leave-One-Out Robustness Checks

Cohort dropped and outcomes	DiD Estimates (95% CI)	P value
**2015 (Oregon)**		
Cannabis	3.23 (2.21-4.25)	p<0.001
Cigarettes	-0.32 (-1.35-0.71)	p=0.54
ENDS	1.22 (0.24-2.20)	p=0.015
**2016 (California and Massachusetts)**		
Cannabis	4.74 (3.50-5.98)	p<0.001
Cigarettes	Main analysis	
ENDS	0.96 (-0.27-2.19)	p=0.13
**California**		
Cannabis	4.63 (3.43-5.83)	p<0.001
Cigarettes	-0.69 (-1.90-0.52)	p=0.26
ENDS	0.99 (-0.19-2.19)	p=0.099
**Massachusetts**		
Cannabis	3.35 (2.34-4.35)	p<0.001
Cigarettes	-0.64 (-1.67-0.38)	p=0.22
ENDS	1.09 (0.13-2.06)	p=0.027
**2017 (Maine and Nevada)**		
Cannabis	3.40 (2.39-4.41)	p<0.001
Cigarettes	-0.38 (-1.41-0.65)	p=0.47
ENDS	1.05 (0.08-2.01)	p=0.034
**2018 (Michigan)**		
Cannabis	2.30 (1.21-3.38)	p<0.001
Cigarettes	-0.68 (-1.81-0.44)	p=0.23
ENDS	0.31 (-0.74-1.36)	p=0.56
**2020 (Arizona and Illinois)**		
Cannabis	3.28 (2.20-4.35)	p<0.001
Cigarettes	-0.30 (-1.37-0.77)	p=0.59
ENDS	1.60 (0.57-2.62)	p=0.002
**2021 (Connecticut, Montana, New Jersey, New York, Virginia)**		
Cannabis	3.48 (2.41-4.55)	p<0.001
Cigarettes	-0.38 (-1.42-0.67)	p=0.48
ENDS	1.45 (0.42-2.48)	p=0.006

© 2025 Hyatt AS et al. *JAMA Network Open*.

Event studies: Drop California

Cannabis

Year.	ATT	p	[95% conf. interval]	
-5	-.0105178	0.457	-.0382342	.0171986
-4	-.0121903	0.334	-.0369237	.0125431
-3	-.0026265	0.801	-.0230551	.017802
-2	-.0013811	0.878	-.0189591	.0161969
-1	0 (reference)			
0	.0167679	0.011	.0039215	.0296142
1	.035299	0.000	.0221904	.0484076
2	.0449186	0.008	.011688	.0781492
3	.0688009	0.000	.0431854	.0944165
4	.0747942	0.000	.0484687	.1011197
5	.0533166	0.038	.002997	.1036362

ENDS

Year.	ATT	p	[95% conf. interval]	
-5	.0181682	0.280	-.0148077	.0511441
-4	.0093675	0.525	-.0195177	.0382527
-3	-.0006828	0.955	-.0243406	.022975
-2	.016631	0.079	-.0019118	.0351738
-1	0 (reference)			
0	-.00662	0.339	-.0201808	.0069407
1	-.0008181	0.907	-.0145206	.0128844
2	.0042367	0.828	-.0340633	.0425367
3	.0535215	0.000	.0287554	.0782876
4	.0337157	0.008	.0086931	.0587382
5	.0029177	0.888	-.0376616	.043497

© 2025 Hyatt AS et al. *JAMA Network Open*.

Event studies: drop Michigan

Cannabis

Year	ATT	p	[95% conf. interval]	
-5	-.0269967	0.290	-.0770101	.0230167
-4	-.0294666	0.239	-.0784894	.0195562
-3	-.0145588	0.302	-.0422099	.0130922
-2	.004775	0.483	-.0085519	.0181019
-1	0 (reference)			
0	.0129551	0.014	.0026551	.0232551
1	.0214701	0.001	.0092489	.0336913
2	.0342248	0.014	.0069479	.0615017
3	.0220377	0.049	.0001	.0439754
4	.025658	0.178	-.0116773	.0629934
5	.0145541	0.192	-.0072888	.0363971

ENDS

Year.	ATT	p.	[95% conf. interval]	
-5	-.0017131	0.952	-.0571323	.053706
-4	-.0097156	0.722	-.0631399	.0437087
-3	.015126	0.369	-.0179019	.0481538
-2	.0060739	0.434	-.0091322	.02128
-1	0 (reference)			
0	.0026627	0.634	-.0082827	.013608
1	.0071487	0.268	-.005502	.0197994
2	.0098477	0.510	-.0194201	.0391155
3	.0084658	0.429	-.0125193	.0294509
4	-.0198221	0.300	-.0572718	.0176275
5	.0000359	0.997	-.0196059	.0196777

© 2025 Hyatt AS et al. *JAMA Network Open*.
