# Supplementary material for: Use of Tobacco and Cannabis Following State-Level Cannabis Legalization
Source: JAMA Netw Open. 2025 Jul 11;8(7):e2520093. doi: 10.1001/jamanetworkopen.2025.20093 (PMC12254894; doi:10.1001/jamanetworkopen.2025.20093)
Supplement: Supplement 2. — Data Sharing Statement [file jamanetwopen-e2520093-s002.pdf]

Data Sharing Statement

Hyatt. Use of Tobacco and Cannabis Following State-Level Cannabis Legalization. *JAMA Netw Open*. Published July 11, 2025. doi:10.1001/jamanetworkopen.2025.20093

Data
Data available: No

Additional Information
Explanation for why data not available: Restricted use file PATH data are available via application from the ICPSR at the University of Michigan. Analytic code is available at https://osf.io/6q87w
